# SIRT5 Promotes Cisplatin Resistance in Ovarian Cancer by Suppressing DNA Damage in a ROS-Dependent Manner via Regulation of the Nrf2/HO-1 Pathway

**DOI:** 10.3389/fonc.2019.00754

**Published:** 2019-08-13

**Authors:** Xiaodan Sun, Shouhan Wang, Junda Gai, Jingqian Guan, Ji Li, Yizhuo Li, Jinming Zhao, Chen Zhao, Lin Fu, Qingchang Li

**Affiliations:** ^1^Department of Pathology, College of Basic Medical Sciences, China Medical University, Shenyang, China; ^2^Department of Hepatopancreatobiliary Surgery, Jilin Province Cancer Hospital, Changchun, China; ^3^Department of Pathology, Carver College of Medicine, University of Iowa, Iowa City, IA, United States; ^4^Department of Pathology, The First Affiliated Hospital, China Medical University, Shenyang, China

**Keywords:** ovarian cancer, SIRT5, Nrf2/HO-1, reactive oxygen species, drug resistance

## Abstract

Sirtuin 5 (SIRT5), a mitochondrial class III NAD-dependent deacetylase, plays controversial roles in tumorigenesis and chemoresistance. Accordingly, its role in ovarian cancer development and drug resistance is not fully understood. Here, we demonstrate that SIRT5 is increased in ovarian cancer tissues compared to its expression in normal tissues and this predicts a poor response to chemotherapy. SIRT5 levels were also found to be higher in cisplatin-resistant SKOV-3 and CAOV-3 ovarian cancer cells than in cisplatin-sensitive A2780 cells. Furthermore, this protein was revealed to facilitate ovarian cancer cell growth and cisplatin-resistance *in vitro*. Mechanistically, we show that SIRT5 contributes to cisplatin resistance in ovarian cancer by suppressing cisplatin-induced DNA damage in a reactive oxygen species (ROS)-dependent manner via regulation of the nuclear factor erythroid 2-related factor 2 (Nrf2)/heme oxygenase 1 (HO-1) pathway.

## Introduction

SIRT5 is a unique member of the Sirtuin family (Sirt1–7), which possesses multiple enzymatic activities including NAD-dependent histone deacetylase (1), potent lysine demalonylase, lysine desuccinylase (2), and lysine glutarylase (3) activities. These specific enzymatic activities indicate that SIRT5 plays a crucial role in regulating multiple cellular metabolic processes such as glycolysis, the tricarboxylic acid cycle, fatty acid oxidation, nitrogen metabolism, and the pentose phosphate pathway (4, 5). In addition, certain aspects of cancer biology, such as stress responses (6, 7), apoptosis (8, 9), and autophagy (10, 11), can be regulated by SIRT5. Moreover, altered cellular metabolism has been recently identified as a hallmark of malignancy and emerging literature suggests that SIRT5 is involved in oncogenesis. For example, either the mRNA or protein expression of SIRT5 was found to be increased in non-small cell lung cancer (NSCLC) (12, 13), hepatocellular carcinoma (HCC) (14), colorectal cancer (CRC) (15), Waldenstrom macroglobulinemia (16), and breast cancer (9, 17), compared to the levels in matched normal tissues. Janus-faced roles of SIRT5 in cancer have also been described. Specifically, the downregulation of SIRT5 was observed in head and neck squamous cell carcinoma (18), liver cancer (19), and endometrial carcinoma (20), which highlight the tumor-suppressive role of SIRT5. Furthermore, controversial roles for SIRT5 in drug resistance have been reported. SIRT5 facilitates NSCLC resistance to cisplatin, 5-fluorouracil (5-FU), and bleomycin (13). Moreover, SIRT5-positive cells in wild-type Kras CRCs were found to be resistant to either chemotherapeutic agents or cetuximab (21). However, a positive association between SIRT5 expression and complete response to neoadjuvant chemotherapy in triple-negative breast cancer patients was previously shown by analyzing data from the Gene Expression Omnibus (GEO) DataSet. In addition, by analyzing the ONCOMINE online database, SIRT5 expression levels were found to be higher in chemotherapy-responders than in non-responders (17). Considering these discrepant findings, further studies are needed to explore the functions of SIRT5 in tumorigenesis and chemoresistance.

Globally, ovarian cancer ranked eighth in incidence and seventh in mortality among all cancers in females in 2018 (WHO, http://gco.iarc.fr/today/home). Standard treatment for this disease involves surgery combined with chemotherapy. However, chemoresistance has become a major reason for poor outcomes in ovarian cancer. Although emerging targeted therapies have improved the survival of chemoresistant ovarian cancer patients, their quality of life and overall survival are still limited due to the side effects associated with such drugs, and the 5-year survival rate for advanced-stage ovarian cancer is only 28.9% (22). Therefore, better curative outcomes for ovarian cancer therapeutics are required. Considering the diverse roles of SIRT5 in cancer biology, we speculated that SIRT5 is a therapeutic target for ovarian cancer. To date, the role of this protein in ovarian cancer has not been elucidated. Therefore, in this context, we investigated the expression pattern of SIRT5 in both human ovarian cancer tissues and ovarian cancer cells. Furthermore, we reveal a potential role for SIRT5 in ovarian cancer cell growth and chemoresistance.

## Materials and Methods

### Antibodies and Reagents

The following antibodies were purchased: primary antibodies to SIRT5 (Cell Signaling Technology, Danvers, MA), H2A histone family member X phosphorylated on S139 (γ-H2AX, Cell Signaling Technology), nuclear factor erythroid-2-related factor 2 (Nrf2; Proteintech, Wuhan, China), heme oxygenase 1 (HO-1; Proteintech), manganese-dependent superoxide dismutase (MnSOD)/SOD2 (Proteintech), breast cancer gene 1 (BRCA1, Cell Signaling Technology), histone H3 (Cell Signaling Technology), and β-actin (Cell Signaling Technology); and secondary antibodies, specifically goat anti-rabbit IgG (Proteintech), goat anti-mouse IgG (Proteintech), and tetramethylrhodamine (TRITC)-conjugated secondary antibody (Proteintech).

Cisplatin and ML385 (an Nrf2-specific inhibitor) were purchased from MCE, China. N-acetyl-L-cysteine (NAC), a reactive oxygen species (ROS) scavenger, was purchased from Selleck, China.

### Immunohistochemical Staining

SIRT5 staining was performed on an ovarian cancer and normal tissue microarray (Biomax, USA), which contained 90 tumor tissues and 10 normal ovarian tissues. Immunostaining was performed based on the ABC immunostaining protocol (MaiXin, Fuzhou, China). Two independent investigators scored the microarray by evaluating the staining intensity and percentage of stained cells blindly and randomly. The staining intensity was scored from 0 to 3 as follows: 0 (negative), 1 (weak), 2 (moderate), and 3 (strong). The percentage of positively stained cells was scored from 0 to 4 as follows: 0 (0%), 1 (1–25%), 2 (26–50%), 3 (51–75%), and 4 (76–100%). The final multiplied scores ranged from 0 to 12 and SIRT5 expression was regarded as positive if the final score was ≥ 6.

### Cell Culture and Transfection

A2780, SKOV-3, and CAOV-3 human ovarian cancer cell lines were used in this study. A2780 and CAOV-3 cells were cultured in DMEM supplemented with 10% fetal bovine serum. SKOV-3 cells were cultured in RPMI-1640 medium supplemented with 10% fetal bovine serum. These cells were grown at 37°C in a humidified atmosphere with 5% CO_2_.

Transient transfection was carried out using Lipofectamine 3000 reagent (Invitrogen) according to the manufacturer's instructions. A SIRT5 expression plasmid (OriGene, Rockville, MD, USA) and the corresponding empty plasmid (OriGene) were used for SIRT5 overexpression and as a negative control, respectively. Cells were transfected with SIRT5-siRNA and negative control siRNA (GenePharma, Shanghai, China) for SIRT5 knockdown experiments.

The cells were pretreated with 5 mM NAC for 2 h to inhibit ROS generation. ML385 was added to cells at a concentration of 5 μM for 48 h in the presence of cisplatin to block the Nrf2 pathway.

### Cell Proliferation and Cell Viability Assays

Cell Counting Kit-8 (CCK-8) assays were performed to assess cell proliferation and cell viability *in vitro*. For cell proliferation, the cells were seeded in 96-well plates at a density of 3,000 cells/well and incubated for 5 days. For cell viability, the cells were seeded in 96-well plates at a density of 5,000 cells/well and treated with the indicated concentration of cisplatin for 48 h or 5 days, changing the cisplatin-containing medium every 2 days. Next, 10 μl of CCK-8 reagent (Beyotime, Shanghai, China) was added to each well, and the cells were incubated for 2 h. The absorbance value (OD) of each well was measured at 450 nm and the cell viability was calculated as follows: cell viability (%) = experimental group OD value / control group OD value × 100%. IC_50_ values (50% inhibition of surviving fraction) were calculated using GraphPad Prism 7.0 software.

### Colony Formation Assays

Cells were plated in six-well culture plates at 500 cells/well. After incubation for 14 days at 37°C in a humidified atmosphere at 5% CO_2_, the cells were washed three times with PBS and stained with Giemsa solution. The number of colonies containing ≥ 50 cells was then counted under a microscope. Colony formation efficiency was calculated as colony numbers / 500 × 100%.

### ROS Detection and Measurement of Intracellular Glutathione (GSH)

The ROS levels induced by cisplatin in ovarian cancer cells were detected using the probe 2′,7′-dichlorodihydrofluorescein diacetate (DCFH-DA, Beyotime), which can be oxidized by intracellular oxygen to dichlorofluorescein, a highly fluorescent compound. After exposure to the indicated concentration of cisplatin for 2, 6, 24, or 48 h, the cells were incubated with a final concentration of 10 μM DCFH-DA in the dark for 20 min at 37°C in a humidified atmosphere at 5% CO_2_, after which the cells were washed three times with cold PBS to remove excess fluorescent probe. The cells were then resuspended in 300 μl of PBS and assessed for fluorescence intensity using a flow cytometer (LSRFortessa, BD Biosciences). The data were analyzed using FlowJo X 10.0.7 Software.

Intracellular GSH levels were measured using a Total Glutathione Assay Kit (Beyotime) according to the manufacturer's instructions. Briefly, the ovarian cancer cells were harvested and lysed in the protein removal solution S provided in the kit. After incubation for 5 min at 4°C, the samples were centrifuged at 10,000 g for 10 min at 4°C. The supernatant was treated with assay solution for 5 min at 25°C and the absorbance at 412 nm was measured using a microplate reader (SpectraMax i3x, Molecular Devices, Sunnyvale, CA). Intracellular GSH levels were quantified by interpolation on standard curves and relative GSH levels were calculated by normalization to the values obtained from A2780 cells.

### Immunofluorescence Staining

Cells were plated in 20-mm culture plates, pretreated with the indicated concentrations of cisplatin for 24 h to observe γ-H2AX foci formation, washed with PBS three times, fixed with 4% paraformaldehyde for 15 min, and permeabilized in 0.1% Triton X-100 for 5 min. After blocking with 5% bovine serum albumin for 1 h at room temperature, the cells were incubated with primary antibodies against γ-H2AX (dilution 1:200), SIRT5 (dilution 1:200), or Nrf2 (dilution 1:200) overnight at 4°C. Then, TRITC-conjugated secondary antibody (dilution 1:1,000) was incubated with the cells for 2 h in the dark at room temperature, and the cells were stained with 4′,6-diamidino-2-phenylindole (DAPI) for 5 min to visualize their nuclei. Images were captured using a fluorescence microscope or an Olympus FV1000 confocal laser-scanning microscope (Olympus, Tokyo, Japan). For quantification of γ-H2AX foci, 5 random fields of cells from each slide were quantified by ImageJ software and foci containing ≥5 cells were considered positive.

### Western Blotting and Isolation of Cytosolic and Nuclear Cell Fractions

Total protein was isolated from SIRT5 overexpressing or knockdown ovarian cancer cells and their corresponding controls, with or without cisplatin treatment. The cells were washed with ice-cold PBS three times and lysed in lysis buffer supplemented with a cocktail of proteinase inhibitors (MCE). Equal amounts of protein (60 μg) from cell extracts were separated by 10% SDS-PAGE and transferred to 0.45- or 0.22-μm (the latter for γ-H2AX) polyvinylidene fluoride (PVDF) membranes (Millipore, Billerica, MA, USA). Following blocking with 5% fat-free milk for 2 h at room temperature, the membranes were incubated with primary antibodies (anti-SIRT5, anti-actin, anti-Nrf2, anti-HO-1, anti-MnSOD/SOD2, anti-BRCA1, anti-histone H3, or anti-γ-H2AX; dilution, 1:1,000) in blocking buffer overnight at 4°C. Then, the membranes were washed three times in Tris-buffered saline with Tween 20 and incubated with secondary antibodies at a dilution of 1:2,500 for 2 h at 37°C. Immunoreactivity was detected using ECL (Thermo Fisher Scientific, Waltham, MA, USA) with a BioImaging System (UVP Inc., Upland, CA, USA). ImageJ software was used to evaluate the gray value of each band.

For cytosolic and nuclear isolation, a Nuclear and Cytoplasmic Protein Extraction kit (Beyotime) was used, following the manufacturer's instructions. Briefly, the collected cells were suspended in ice-cold hypotonic buffer and incubated on ice for 20 min. The extracts were then centrifuged at 12,000 g for 5 min, and the supernatants were collected as cytosolic fractions. The pellets were washed with ice-cold PBS and resuspended in lysis buffer, followed by vortexing at the highest speed. These extracts were centrifuged at 12,000 g for 10 min, and the supernatants were collected as nuclear fractions.

### Gene Expression Profiling Interactive Analysis (GEPIA) Database and Statistical Analysis

The GEPIA database (http://gepia.cancer-pku.cn/), a newly developed web-based tool, provides tumor vs. normal differential expression analysis, correlation analysis based on the Cancer Genome Atlas, and genotype-tissue expression data. It was used to analyze the expression of SIRT5, Nrf2, and HO-1 in ovarian cancer and normal tissues and the correlation between SIRT5 and Nrf2, MnSOD, and BRCA1. All experiments were repeated at least three times and the data were expressed as the means ± standard deviations. Statistical analysis was performed with GraphPad Prism 7.0 software. Statistical significance was determined based on a Student's *t*-test or one-way ANOVA. The χ^2^ test was used to determine the correlation between SIRT5 expression and clinicopathologic characteristics. *P* < 0.05 was considered to denote a statistically significant difference.

## Results

### SIRT5 Expression Is Increased in Ovarian Cancer Tissues and High SIRT5 Levels Predict Poor Chemotherapy Response

**Figure 1:**
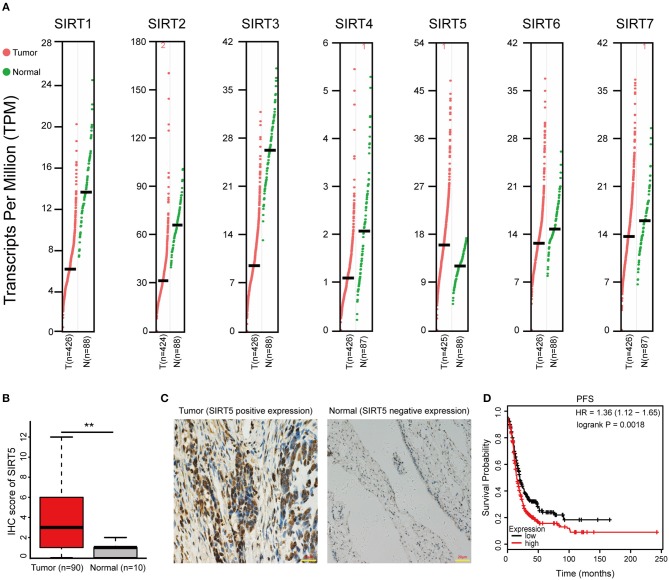
SIRT5 expression is increased in ovarian cancer tissues and high SIRT5 level predicts poor chemo-response. **(A)** The mRNA expression of *Sirtuins* in patients with ovarian cancer (GEPIA). **(B)** Immunohistochemical staining results of SIRT5 expression in 90 cases ovarian cancer tissues and 10 cases normal tissues microarrays. **(C)** Representative immunohistochemical staining images of SIRT5 expression (positive or negative) in ovarian cancer and normal tissues. (Magnification, × 400; Scale bar = 20 μm). **(D)** High SIRT5 level predicts poor progression-free survival (PFS) by online Kaplan-Meier analysis. ^**^P < 0.01.

First, the GEPIA database was used to compare the mRNA expression of seven *Sirtuin* members between ovarian cancer and normal tissues. Only the mRNA level of *SIRT5* was higher, while that of other isoforms was lower, in tumors than it was in normal tissues (Figure 1A). Then, immunohistochemistry was performed to verify this result. SIRT5 was more highly expressed in ovarian cancer tissues than in normal tissues and was mainly localized to the cytoplasm (Figures 1B,C). In addition, as shown in Table 1, higher SIRT5 levels were positively correlated with advanced International Federation of Gynecology and Obstetrics (FIGO) stage (*P* < 0.0001) and lymph node metastasis (*P* = 0.0019), but negatively correlated with a low grade of differentiation (*P* = 0.0179). The prognostic value of SIRT5 was then determined by Kaplan–Meier analysis using the KM plotter online software (http://kmplot.com/analysis/) based on 614 ovarian cancer patients who received chemotherapy. To investigate whether SIRT5 is relevant to chemoresistance, progression-free survival (PFS) was chosen as the primary endpoint, and patients with high SIRT5 expression exhibited significantly shorter PFS than those with low expression (*P* = 0.0018; Figure 1D). In summary, SIRT5 expression is increased in ovarian cancer tissues and high SIRT5 levels predict poor chemotherapy response.

**Table 1:** Association of SIRT5 expression with clinicopathological characteristics of ovarian cancer tissue microarrays.

**Clinicopathological characteristics**	**Total number**	**SIRT5 negative or weak expression**	**SIRT5 positive expression**	***P* value**
Age				0.0786
<60	73	47 (64.38%)	7 (35.62%)	
≥60	17	7 (41.18%)	10 (58.82%)	
Histology				0.085
Serous carcinoma	72	40 (55.56%)	32 (44.44%)	
Mucinous adenocarcinoma+others	18	14 (77.78%)	4 (22.22%)	
Differentiation				**0.0179**
High grade	65	34 (52.31%)	31 (47.69%)	
Low grade	25	20 (80.00%)	5 (20.00%)	
Figo stage				**<0.0001**
I	47	44 (93.62%)	3 (6.38%)	
II-IV	43	10 (23.26%)	33 (76.74%)	
Lymph node metastasis				**0.0019**
Negative	68	47 (69.12%)	21 (30.88%)	
Positive	22	7 (31.82%)	15 (68.18%)	

*The bold fonts represent the significant values*.

### SIRT5 Expression Is Increased in Cisplatin-Resistant Ovarian Cancer Cells and Cisplatin Upregulates SIRT5 Levels

**Figure 2:**
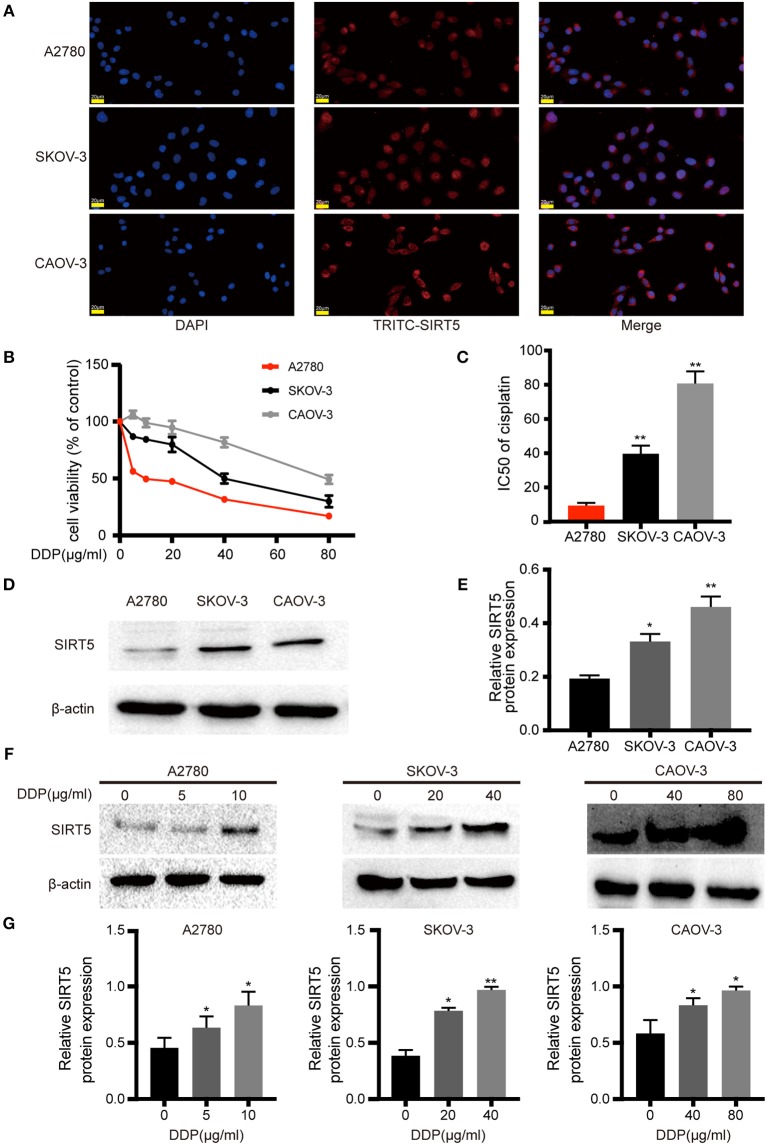
SIRT5 expression is involved in cisplatin-resistant of ovarian cancer cells. **(A)** SIRT5 expression in three ovarian cancer cell lines analyzed by immunofluorescence staining using TRITC-labeled antibodies; nuclei were stained with DAPI. **(B)** Differences in cisplatin sensitivity of three ovarian cancer cell lines assessed by CCK-8 assay. **(C)** Calculated IC50 values after cells were exposed to the indicated dose of cisplatin for 48 h. **(D)** SIRT5 protein levels in three ovarian cancer cell lines assessed by western blot and **(E)** relative protein expression was quantified by relative gray value of bands with ImageJ software. **(F,G)** SIRT5 protein levels were upregulated during exposed to indicated dose of cisplatin in three ovarian cancer cell lines for 48 h. DDP, cisplatin. Data are presented as the mean ± SD of three independent experiments. Scale bar = 20 μm, ^*^P < 0.05, ^**^P < 0.01, compared with A2780 or control cells.

Based on the aforementioned results, the expression pattern of SIRT5 in ovarian cancer cells was assessed. First, immunofluorescence staining indicated that this marker was localized mainly to the cytoplasm of A2780, SKOV-3, and CAOV-3 cells (Figure 2A), in agreement with the immunohistochemistry staining of tissues. Western blotting of cytosolic and nuclear fractions verified this result (Figure S1A). Then, the sensitivities to cisplatin among the three ovarian cancer cell lines were compared. The cells were treated with different concentrations of cisplatin (0–80 μg/ml) for 24 h and different degrees of sensitivity to cisplatin were noted. SKOV-3 (IC_50_ = 39.743 ± 4.756 μg/ml) and CAOV-3 (IC_50_ = 80.813 ± 7.058 μg/ml) cell lines were less sensitive to cisplatin than A2780 cells (IC_50_ = 9.362 ± 0.489 μg/ml; Figures 2B,C). These results are consistent with previous descriptions of these three ovarian cancer cell lines (23–25).

SIRT5 expression was higher in SKOV-3 and CAOV-3 cisplatin-resistant cells than in A2780 cisplatin-sensitive cells (Figures 2D,E). Then, whether cisplatin could affect the protein levels of SIRT5 was investigated. The three ovarian cancer cell lines were incubated without cisplatin, with 50% of the IC_50_ dose, and with the IC_50_ dose of cisplatin for 24 h. SIRT5 expression was significantly upregulated in the cisplatin-treated cells in a concentration-dependent manner compared to control cells (Figures 2F,G). Taken together, these results reveal a causal relationship between SIRT5 expression and cisplatin resistance in ovarian cancer cells.

### SIRT5 Promotes Cell Proliferation and Cisplatin Resistance in Ovarian Cancer Cells

**Figure 3:**
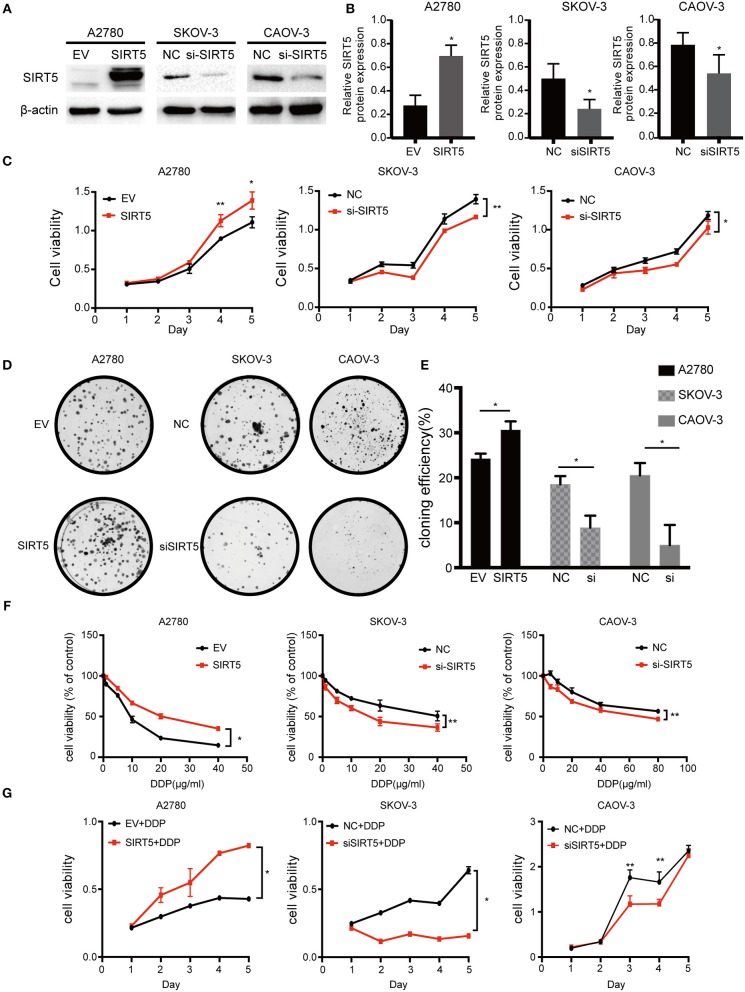
SIRT5 promotes cell proliferation and cisplatin resistance in ovarian cancer cells. **(A)** A2780 cell lines were transfected with SIRT5 plasmid and SKOV-3 and CAOV-3 cell lines were transfected with SIRT5-siRNA. The transfection efficiency was detected by western blot and **(B)** the relative gray values were shown in histogram. **(C)** Overexpression of SIRT5 in A2780 cells promoted cell growth and knockdown of SIRT5 inhibited cell growth in SKOV-3 and CAOV-3 cells. After reated with indicated dose of cisplatin, the relative cell numbers were evaluated with CCK-8 assay at the indicated time points. **(D)** A significant increase in colony number was observed in A2780 cells with SIRT5 overexpression and an opposite trend was observed in SKOV-3 and CAOV-3 cells with knockdown of SIRT5. **(E)** Colony numbers were counted under microscope and the colony formation efficiency was calculated. **(F)** IC50 of cisplatin was increased in A2780 cells with SIRT5 overexpression and decreased in SKOV-3 and CAOV-3 cells with knockdown of SIRT5. **(G)** The transfected cells were treated with IC50 dose of cisplatin for consecutive 5 days respectively. Cell viability was assessed by CCK-8 assay. Data are presented as the mean ± SD of three independent experiments. EV, empty vector. NC, negative control. DDP, cisplatin. ^*^P < 0.05, ^**^P < 0.01, compared with the EV or NC cells.

As SIRT5 was hypothesized to play a role in ovarian cancer development and chemoresistance, its effect on the biological behaviors of the three cell lines was investigated. Based on its basal protein levels, SIRT5 was either overexpressed or silenced. A2780 cells were transfected with a SIRT5 overexpression plasmid or empty vector, while knockdown of SIRT5 in SKOV-3 and CAOV-3 cells was achieved by transfecting them with SIRT5-siRNA or negative control siRNA (Figures 3A,B). The results indicated that SIRT5 overexpression could significantly promote A2780 cell proliferation and colony formation, while the opposite effect was observed in SKOV-3 and CAOV-3 cells upon SIRT5 knockdown (Figures 3C–E).

Next, cell viability assays were performed to investigate the relationship between SIRT5 and cisplatin resistance. The transfected cell lines were treated with increasing concentrations of cisplatin for 24 h to observe the effect on cisplatin IC_50_ values. Moreover, these transfected cells were treated with their respective IC_50_ dose of cisplatin for 5 consecutive days. The results showed that the IC_50_ values and cell viability were elevated after overexpression of SIRT5 in A2780 cells, whereas both values were decreased upon SIRT5 downregulation in SKOV-3 and CAOV-3 cells (Figures 3F,G). These findings suggest that SIRT5 promotes proliferation and cisplatin resistance in ovarian cancer cells.

### SIRT5 Suppresses Cisplatin-Induced DNA Damage

**Figure 4:**
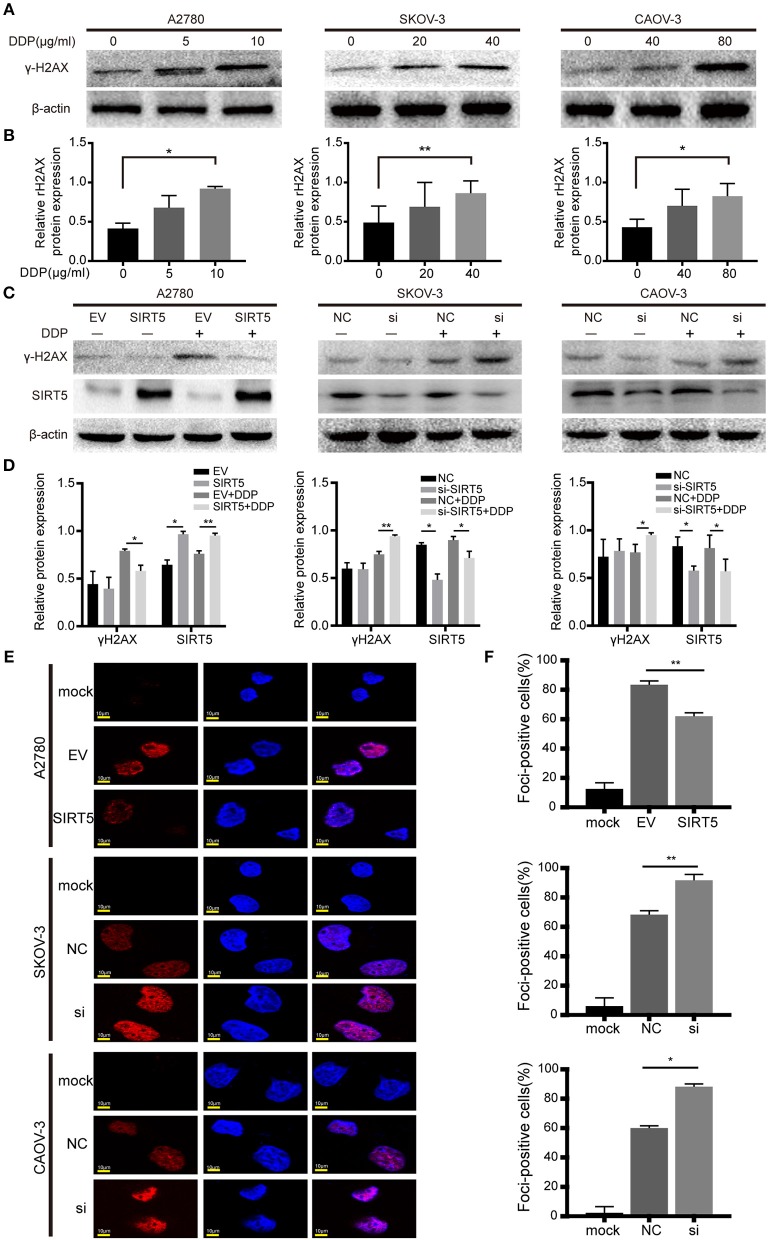
SIRT5 suppresses cisplatin induced DNA damage. **(A)** The γ-H2AX protein levels were upregulated during exposure to cisplatin in three ovarian cancer cells assessed by western blot and **(B)** the relative gray values were shown in histogram. **(C)** The transfected cells were treated with or without IC50 dose of cisplatin for 24 h. The γ-H2AX protein levels were assessed by western blot and **(D)** the relative gray values were shown in histogram. **(E)** The γ-H2AX foci formation was observed by immunofluorescence staining. The mock cells were untreated and the transfected cells were treated with IC50 dose of cisplatin for 24 h respectively. **(F)** The quantification results of γ-H2AX foci in three ovarian cancer cells. Data are presented as the mean ± SD of three independent experiments. EV, empty vector. NC, negative control. DDP, cisplatin. Scale bar = 10 μm, ^*^P < 0.05, ^**^P < 0.01.

As mentioned above, SIRT5 was found to promote resistance to cisplatin in ovarian cancer cells, raising the question of how SIRT5 participates in the induction of chemoresistance. First, we confirmed that cisplatin can lead to the accumulation of γ-H2AX protein, which is a marker of DNA double-strand breaks, in a concentration-dependent manner (Figures 4A,B). Moreover, cisplatin-induced DNA damage could be suppressed by the overexpression of SIRT5 in A2780 cells, whereas γ-H2AX protein levels were increased upon SIRT5 downregulation in SKOV-3 and CAOV-3 cells (Figures 4C,D). In addition, immunofluorescence validated that SIRT5 overexpression could inhibit the formation of γ-H2AX foci, whereas this suppressive effect was abrogated upon SIRT5 knockdown (Figures 4E,F). Therefore, SIRT5 was shown to promote cisplatin resistance in ovarian cancer cells by inhibiting DNA damage.

### SIRT5 Eliminates Cisplatin-Induced ROS to Reduce DNA Damage

**Figure 5:**
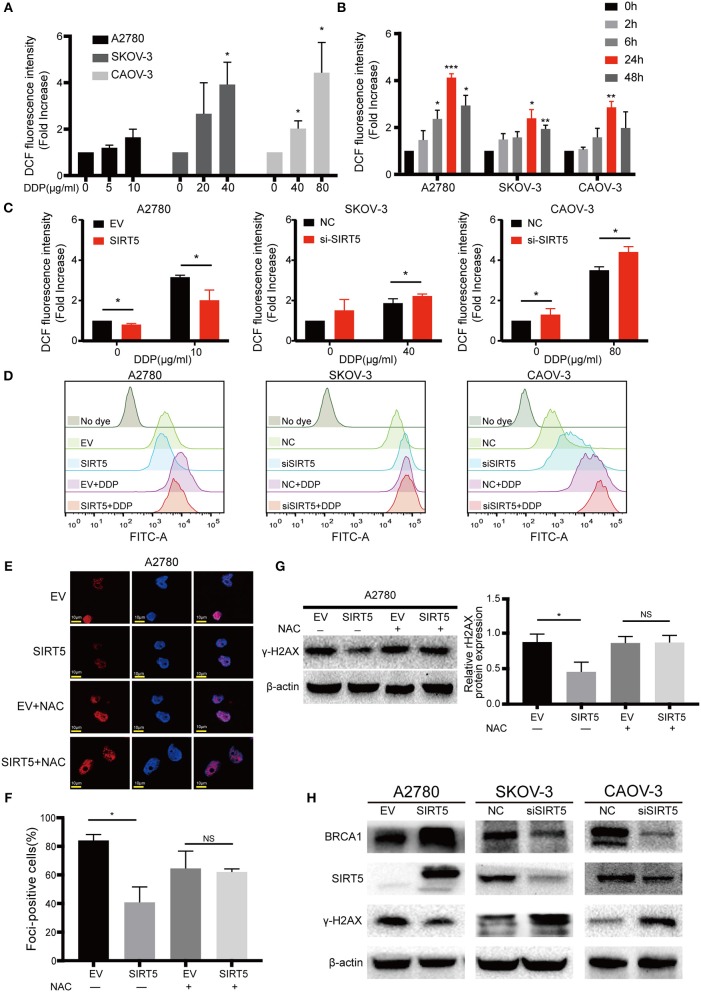
SIRT5 eliminates cisplatin induced ROS to reduce DNA damage. **(A)** Cisplatin induced ROS generation in concentration and **(B)** time dependent manner in three ovarian cancer cells. The cells were treated with indicated dose of cisplatin and collected at indicated time points. The ROS levels were measured by flow cytometry and 0 h served as a control group. **(C)** Overexpression of SIRT5 inhibited ROS production and knockdown of SIRT5 attenuated the inhibition effect. **(D)** Representative flow cytometry results analyzed by FlowJo software. **(E,F)** NAC, a ROS scavenger, reversed the inhibition of cisplatin induced DNA damage in A2780 cells with overexpression SIRT5 by assessing γ-H2AX foci formation and **(G)** γ-H2AX protein levels. Cells were pretreated with 5 mM NAC for 2 h to inhibit ROS generation and then exposure to cisplatin for 24 h. **(H)** The levels of γ-H2AX was suppressed when BRCA1 was upregulated upon overexpression of SIRT5, and increased after downregulated of BRCA1 upon knockdown of SIRT5. Data are presented as the mean ± SD of three independent experiments. EV, empty vector; NC, negative control; DDP, cisplatin; NS, not significant. Scale bar = 10 μm, ^*^P < 0.05, ^**^P <0.01, ^***^P <0.001.

Next, the mechanism by which SIRT5 suppresses cisplatin-induced DNA damage was investigated. As SIRT5 has been reported to regulate ROS (26) and excessive ROS induced by cisplatin can lead to DNA damage (27–29), we hypothesized that SIRT5 suppresses cisplatin-induced DNA damage by eliminating ROS. First, we confirmed that the levels of intracellular ROS induced by cisplatin were increased in both a concentration- and time-dependent manner. The levels of ROS peaked after 24 h of exposure to cisplatin and decreased by 48 h in all three ovarian cancer cell lines (Figures 5A,B). Therefore, we observed ROS levels in these cell lines at 24 h after cisplatin treatment for the subsequent experiments. Cisplatin-induced ROS levels were significantly inhibited upon overexpression of SIRT5 in A2780 cells, whereas the levels were increased after SIRT5 was silenced in SKOV-3 and CAOV-3 cells (Figures 5C,D). As expected, the inhibition of cisplatin-induced DNA damage by SIRT5 was reversed by the administration of NAC, a ROS scavenger (Figures 5E–G). These results indicate that SIRT5 suppresses cisplatin-induced DNA damage in a ROS-dependent manner in ovarian cancer cells.

In addition, using the GEPIA database, *SIRT5* expression was found to have a positive relationship with *BRCA1* expression, which is a well-known DNA damage repair gene (*P* = 6.6e-7, Figure S1B). In the present study, the levels of γ-H2AX were suppressed when BRCA1 was upregulated upon overexpression of SIRT5, and they were increased after downregulation of BRCA1 upon knockdown of SIRT5 (Figure 5H). These results suggest that SIRT5 also suppresses γ-H2AX expression by positively regulating BRCA1 expression, but the specific mechanism needs to be further explored.

### SIRT5 Inhibits ROS by Positively Regulating the Nrf2/HO-1 Pathway

**Figure 6:**
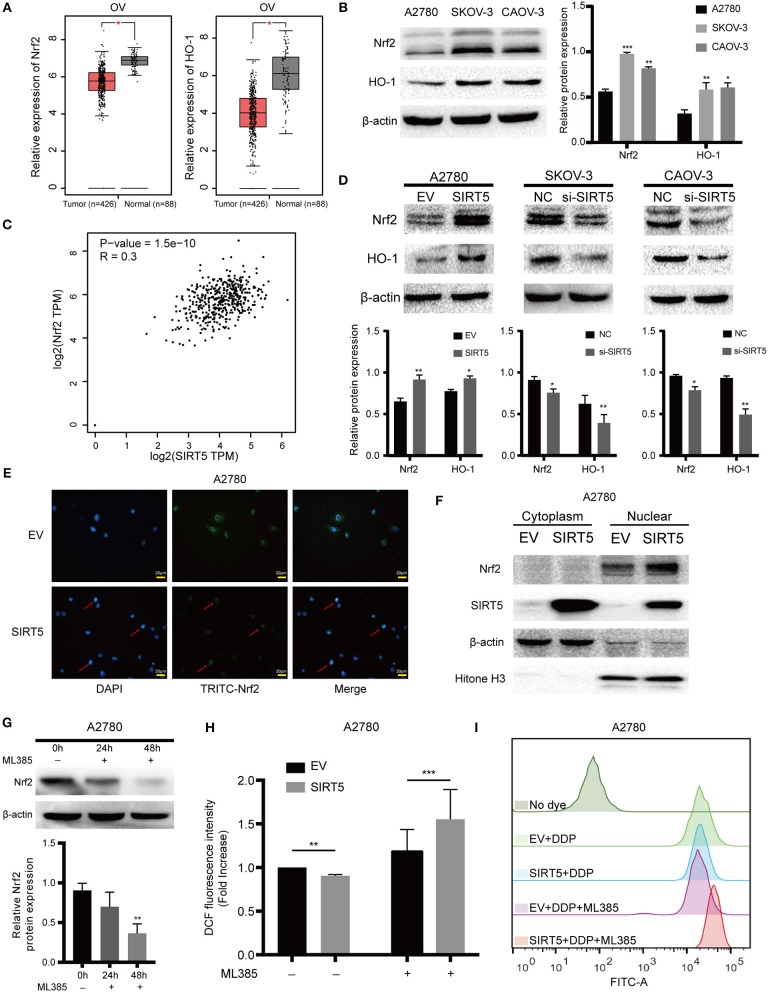
SIRT5 inhibits ROS via positively regulating Nrf2/HO-1 pathway. **(A)** The mRNA expression of *Nrf2* and *HO-1* in ovarian cancer and normal tissues (GEPIA). **(B)** Nrf2 and HO-1 protein levels were higher in SKOV-3 and CAOV-3 than A2780 cells assessed by western blot and the relative gray values were shown in histogram. **(C)** The correlation between *Nrf2* and *SIRT5* in ovarian cancer based on GEPIA database by Pearson correlation coefficient. **(D)** Overexpression of SIRT5 upregulated Nrf2 and HO-1 protein levels and knockdown of SIRT5 reduced their expression assessed by western blot and the relative gray values were shown in histogram. **(E,F)** Overexpression of SIRT5 facilitated the nuclear translocation of Nrf2 by immunofluorescence staining and western blot after nuclear and cytoplasm isolation. **(G)** ML385, a specific inhibitor of Nrf2, was added into A2780 cells with terminal concentration of 5 μM for 24 or 48 h and the inhibition efficiency was measured by western blot. **(H)** The inhibition effect of ROS by overexpression SIRT5 in A2780 cells was reversed when ML385 was applied. **(I)** Representative flow cytometry results analyzed by FlowJo software. Data are presented as the mean ± SD of three independent experiments. EV, empty vector; NC, negative control; DDP, cisplatin; TPM, transcripts per million. Scale bar = 20 μm, ^*^P < 0.05, ^**^P<0.01, ^***^P<0.001, compared with A2780, the EV or NC cells.

As SIRT5 is closely related to the mitochondria, the expression of MnSOD/SOD2, which is a well-known antioxidant enzyme, was measured initially. However, the level of this enzyme was not significantly changed after upregulation or downregulation of SIRT5 and the expression of *SOD2* mRNA had no significant relationship with *SIRT5* mRNA expression in ovarian cancer based on the GEPIA database (Figures S1C,D). Next, whether SIRT5 could inhibit ROS by regulating the Nrf2/HO-1 pathway, which is also known to eliminate ROS (30), was investigated. The mRNA expression levels of *Nrf2* and *HO-1* were lower in ovarian cancer than in normal tissues based on the GEPIA database (Figure 6A). Interestingly, the protein levels of Nrf2 and HO-1 were higher in SKOV-3 and CAOV-3 cells than in A2780 cells (Figure 6B). Moreover, a significant, positive correlation was identified between *Nrf2* and *SIRT5* mRNA expression in ovarian cancer based on the GEPIA database (*P* = 1.5e-10, *R* = 0.3) (Figure 6C). Further, Nrf2 and HO-1 proteins were upregulated upon SIRT5 overexpression in A2780 cells and were downregulated upon SIRT5 silencing in SKOV-3 and CAOV-3 cells (Figure 6D). In addition, overexpression of SIRT5 facilitated the nuclear translocation of Nrf2 by immunofluorescence staining, which was corroborated by western blotting of cytosolic and nuclear fractions (Figures 6E,F). These observations suggest that SIRT5 can enhance the expression of Nrf2 and its target gene HO-1 in ovarian cancer.

To provide further support, ML385, a specific inhibitor of Nrf2, was utilized to treat A2780 cells for 24 or 48 h. As shown in Figure 6G, Nrf2 was significantly suppressed at 48 h. ROS levels in A2780 cells overexpressing SIRT5 were then measured after pretreating the cells with or without ML385 for 48 h and cisplatin for 24 h. The results show that the inhibition of ROS by SIRT5 was reversed upon ML385 treatment (Figures 6H,I). Taken together, SIRT5 can inhibit ROS by positively regulating the Nrf2/HO-1 pathway.

## Discussion

In this study, among the *Sirtuin* family, *SIRT5* was highly expressed in ovarian cancer compared to its expression in normal tissues, based on the GEPIA database and this result was verified by immunohistochemistry. In addition, high levels of SIRT5 predicted shorter PFS and were positively associated with clinicopathologic characteristics of ovarian cancer, such as advanced FIGO stage and lymph node metastasis, but were negatively correlated with differentiation. These results reveal a potential prognostic role for SIRT5 in ovarian cancer patients. Consistent with our results, SIRT5 was shown to be overexpressed in human NSCLC (13), triple-negative breast cancer, breast cancer with BRCA1 mutation subtypes (17), CRC (15), and HCC (14). Further statistical analysis showed that higher SIRT5 was significantly associated with malignant tumor characteristics such as larger tumor size, lymph node metastasis, advanced TNM stage, and poor survival. Besides, a shorter time to post-therapeutic recurrence in wild-type Kras CRC patients was found to correlate with high expression of SIRT5 (21). However, upregulated SIRT5 in liver cancer tissues was found to be associated with favorable prognosis (19). Regarding mRNA or protein levels of SIRT5, Janus-faced expression has been identified. For example, SIRT5 was overexpressed in the aforementioned tumors and B cell malignancies (16), but was decreased in endometrial carcinoma (20) and head and neck squamous cell carcinoma (18).To summarize, SIRT5 might act as either a tumor promoter or suppressor, in a context-specific manner.

Although SIRT5 is predominantly a mitochondrial matrix protein, possessing a well-defined mitochondrial localization sequence (31), it was localized to both the cytoplasm and nucleus in three ovarian cancer cell lines, but the majority of SIRT5 was found in the cytoplasm in our study, which is consistent with other reports (32–34). Interestingly, nuclear and cytosolic SIRT5 in cerebellar granule neurons exerted a protective effect for cells, whereas mitochondrial SIRT5 promoted neuronal death (34). However, the mechanism by which SIRT5 localization contributes to these effects remains unknown.

Additionally, we revealed that SIRT5 expression was higher in SKOV-3 and CAOV-3 cells, which were shown to be resistant to cisplatin in our study, than in A2780 cells, which were confirmed to be sensitive to cisplatin (23–25). Moreover, SIRT5 expression was upregulated by cisplatin in a concentration-dependent manner. Similarly, the significant enrichment of endogenous SIRT5 protein after exposure to chemotherapeutic agents or cetuximab was confirmed previously in two wild-type Kras CRC cell lines (21). Consequently, we speculated that SIRT5 is involved in the progression and chemoresistance of ovarian cancer. As expected, the overexpression of SIRT5 significantly promoted cell proliferation and cisplatin resistance *in vitro*, while an inhibitory effect was observed upon SIRT5 downregulation. In agreement with our results, SIRT5 was found to drive HEK293 cancer cell proliferation via desuccinylation and activation of the serine hydroxymethyltransferase SHMT2 (35) and promote cell proliferation in HCC by targeting E2F transcription factor 1 (14). In contrast, SIRT5 was found to inhibit gastric cancer cell proliferation and tumor formation by inhibiting aerobic glycolysis (36). Interestingly, SIRT5 is not necessary for BrafV600E-mediated cutaneous melanoma initiation and growth *in vivo* (37). With respect to its function in chemoresistance, SIRT5 knockdown sensitizes NSCLC A549 cells to multiple chemotherapeutics including cisplatin (13). Moreover, SIRT5 was determined to have a partial inhibitory effect on the tumor suppressor SUN2, which was found to increase the sensitivity of lung cancer to cisplatin by inducing apoptosis (12). In wild-type Kras CRCs, SIRT5-positive cells were also shown to be resistant to either chemotherapeutic agents, such as 5-FU and oxaliplatin, or cetuximab (21). In contrast, an analysis of data from the GEO DataSet revealed high levels of *SIRT5* in triple-negative breast cancer patients who showed complete response to neoadjuvant chemotherapy. Analysis of the ONCOMINE database also suggested that SIRT5 expression levels were higher in chemotherapy responders than in non-responders (17). In conclusion, further study is needed to explore the role of SIRT5 in tumorigenesis and chemoresistance.

Cisplatin, one of the most widely applied chemotherapeutic agents for multiple solid tumors including ovarian cancer, was initially described as a DNA intrastrand cross-linker that interacts with DNA to form DNA adducts, resulting in the activation of several signal transduction pathways including those involved in DNA damage repair and apoptosis (38). Recent reports have suggested that a novel cytotoxic effect for most genotoxic drugs including cisplatin is to promote ROS-dependent apoptosis (39, 40) or DNA damage (27–29). Therefore, the activation of a ROS-scavenging mechanism in cancer cells confers resistance to chemotherapy (21). In this study, we found that SIRT5 potently inhibits cisplatin-induced DNA damage, as indicated by γ-H2AX protein levels in western blotting and foci formation in immunofluorescence staining. Similarly, after SIRT5 knockdown, the levels of γ-H2AX were previously shown to be significantly upregulated in both CRCs and HCC (15, 19). Then, we confirmed that cisplatin can induce ROS production in a concentration- and time-dependent manner, and that ROS levels peaked after exposure to an IC_50_ treatment of cisplatin for 24 h. ROS levels induced by cisplatin were significantly reduced upon SIRT5 overexpression but were increased after SIRT5 knockdown. Furthermore, the inhibitory effect of SIRT5 on DNA damage was attenuated when NAC, a ROS scavenger, was applied. These results verified our hypothesis that SIRT5 promotes cisplatin resistance in ovarian cancer by suppressing cisplatin-induced DNA damage in a ROS-dependent manner. In agreement with our results, SIRT5 was found to demalonylate and inactivate succinate dehydrogenase complex subunit A (SDHA) in CRCs, resulting in activation of the ROS-scavenging enzyme, thioredoxin reductase 2 (TrxR2) and finally leading to chemotherapy resistance (21). Likewise, other mechanisms underlying SIRT5-mediated ROS detoxification have been reported. For instance, SIRT5 was identified as a safeguard against oxidative stress-induced apoptosis in cardiomyocytes and neuroblastoma cells (6, 7). In NSCLC, SIRT5 was found to desuccinylate and activate Cu/Zn superoxide dismutase (SOD1) to eliminate ROS when the proteins were co-expressed (41). Moreover, the desuccinylation of isocitrate dehydrogenase 2 (IDH2) or pyruvate kinase M2 (PKM2) and the deglutarylation of glucose-6-phosphate dehydrogenase (G6PD) by SIRT5 leads to the production of sufficient NADPH, a major intracellular reductant, to attenuate cellular ROS levels (42, 43). Interestingly, the expression of MnSOD/SOD2, a known antioxidant enzyme, was not significantly changed after upregulation or downregulation of SIRT5 in our study. Collectively, these studies highlight the fact that SIRT5 promotes ROS detoxification via the post-translational modification of multiple, vital antioxidant enzymes.

Another mechanism of cisplatin resistance is the enhancement of DNA damage repair. *BRCA1*, which is a key gene in the DNA damage repair pathway, was identified as having a positive correlation with *SIRT5* expression in ovarian cancer, based on the GEPIA database. Further, the levels of γ-H2AX were suppressed when BRCA1 was upregulated upon overexpression of SIRT5, and they were increased when BRCA1 was downregulated upon knockdown of SIRT5. These results suggested that SIRT5 also contributes to cisplatin resistance by positively regulating BRCA1 expression, but the exact mechanism needs to be explored further.

In addition to the abovementioned enzymes, transcription factors that promote the expression of antioxidant defense genes, such as forkhead box O3 (FOXO3), could be deacetylated at critical lysine residues by SIRT5, promoting their nuclear localization and leading to decreased ROS levels (44). In our study, another transcription factor, Nrf2, which promotes the expression of antioxidant and detoxifying genes, and HO-1, a key target gene of Nrf2, were overexpressed in cisplatin-resistant SKOV-3 and CAOV-3 cells, in agreement with reports of high levels of Nrf2 and HO-1 in drug-resistant tumor cells (45–47). Interestingly, the mRNA expressions of *Nrf2* and *HO-1* were lower in ovarian cancer than in normal tissues based on the GEPIA database, in contrast to reports of their upregulated protein levels in tumors such as ovarian cancer (48, 49). Moreover, in our study, the Nrf2/HO-1 pathway was found to be positively regulated by SIRT5 and overexpression of SIRT5 facilitated Nrf2 nuclear translocation, consistent with a report that the mRNA levels of *Nrf2* and its downstream target genes are reduced upon SIRT5 knockdown in NSCLC (13). In addition, in the present study, the downregulation of ROS by SIRT5 was reversed when a specific Nrf2 inhibitor, ML385, was utilized. These results provide evidence that SIRT5 inhibits ROS production via positive regulation of the Nrf2/HO-1 pathway. GSH, a known ROS scavenger, was identified previously as mediating resistance to cisplatin (50–53). It was reported that Nrf2 is involved in the regulation of GSH abundance and that Nrf2 activation can result in high GSH dependency of the affected cells (54). Consistent with these reports, in our study, higher levels of GSH were found in SKOV-3 and CAOV-3 cisplatin-resistant cell lines relative to the levels in A2780 sensitive cells (Figure S1E). To summarize, SIRT5 may also function as a ROS inhibitor by activating Nrf2, leading to increased GSH levels, but this hypothesis should be confirmed by future experiments.

The limitation of our study is that the exact mechanism by which SIRT5 functions as a mitochondrial enzyme to regulate the Nrf2/HO-1 pathway was not clarified. However, one possibility is that SIRT5 inhibits autophagic flux via the deacetylation of lactate dehydrogenase B (LDHB) (10), leading to an increase in p62, which competes with Nrf2 for Kelch-like ECH-associated protein 1 (KEAP1) binding (55). This results in Nrf2 dissociation from KEAP1 and prolonged activation of Nrf2 (56, 57). Another possibility is that SIRT5 competes with SIRT2 to interact with Nrf2, which blocks the deacetylation of Nrf2 by SIRT2, leading to an increase in nuclear Nrf2 levels (58, 59). In addition, it was also reported that BRCA1 could interact with Nrf2 and promote its stability and activation (60–62), we hypothesize that SIRT5 actives Nrf2 pathway by positively regulating BRCA1. However, these hypotheses need to be tested further.

**Figure 7:**
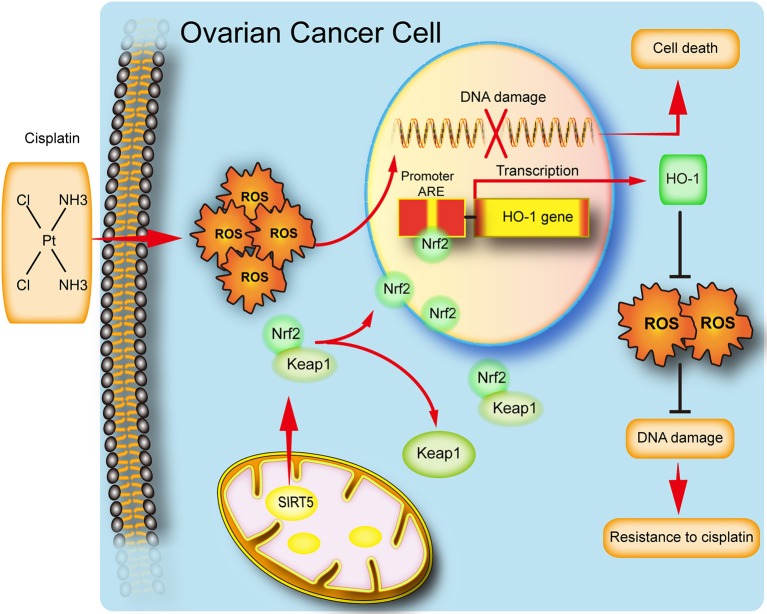
Schematic representation of SIRT5 mediated resistance of ovarian cancer cells to cisplatin.SIRT5 positively regulates Nrf2, resulting in the nuclear translocation of Nrf2. Nuclear Nrf2 binds to antioxidant responsive elements(ARE) and activates HO-1 transcription and thus eliminates cisplatin induced ROS, leading to inhibition of DNA damage and finally cisplatin resistance.

In conclusion, our study implies that SIRT5 expression is increased in ovarian cancer tissues and its high level predicts a poor chemotherapy response. We also reveal a potential function for SIRT5 in ovarian cancer proliferation and chemoresistance. Specifically, SIRT5 contributes to cisplatin resistance by suppressing cisplatin-induced DNA damage in a ROS-dependent manner via the regulation of Nrf2/HO-1 signaling (Figure 7). Therefore, SIRT5 might serve as a prognostic factor for ovarian cancer and SIRT5 inhibitors combined with chemotherapy could represent a novel therapeutic strategy for ovarian cancer patients.

## Data Availability

The raw data supporting the conclusions of this manuscript will be made available by the authors, without undue reservation, to any qualified researcher. Publicly available datasets were also analyzed in this study. This data can be found here: http://gepia.cancer-pku.cn/.

## Author Contributions

QL, LF, CZ, SW, and XS contributed conception and design of the study. XS, SW, JGa, JGu, JL, YL, and JZ performed experiment and the statistical analysis. XS and SW wrote the first draft of the manuscript. All authors contributed to manuscript revision, read and approved the submitted version.

### Conflict of Interest Statement

The authors declare that the research was conducted in the absence of any commercial or financial relationships that could be construed as a potential conflict of interest.

## Supplementary Material

Figure S1 **(A)** The localization of SIRT5 in three ovarian cancer cell lines was validated by western blot after nuclear and cytoplasm isolation. **(B)** *SIRT5* had a positive relationship with *BRCA1* based on GEPIA database. **(C)** MnSOD/SOD2 protein levels were not significantly changed after upregulation or downregulation of SIRT5 levels. **(D)** *SIRT5* had no significant relationship with *MnSOD/SOD2* based on GEPIA database. **(E)** The relative levels of glutathione (GSH) in three ovarian cancer cells. TPM, transcripts per million. ^*^*P* < 0.05.
