# Supplementary figures and images for: SIRT5 Promotes Cisplatin Resistance in Ovarian Cancer by Suppressing DNA Damage in a ROS-Dependent Manner via Regulation of the Nrf2/HO-1 Pathway
Source: Front Oncol. 2019 Aug 13;9:754. doi: 10.3389/fonc.2019.00754 (PMC6700301; doi:10.3389/fonc.2019.00754)

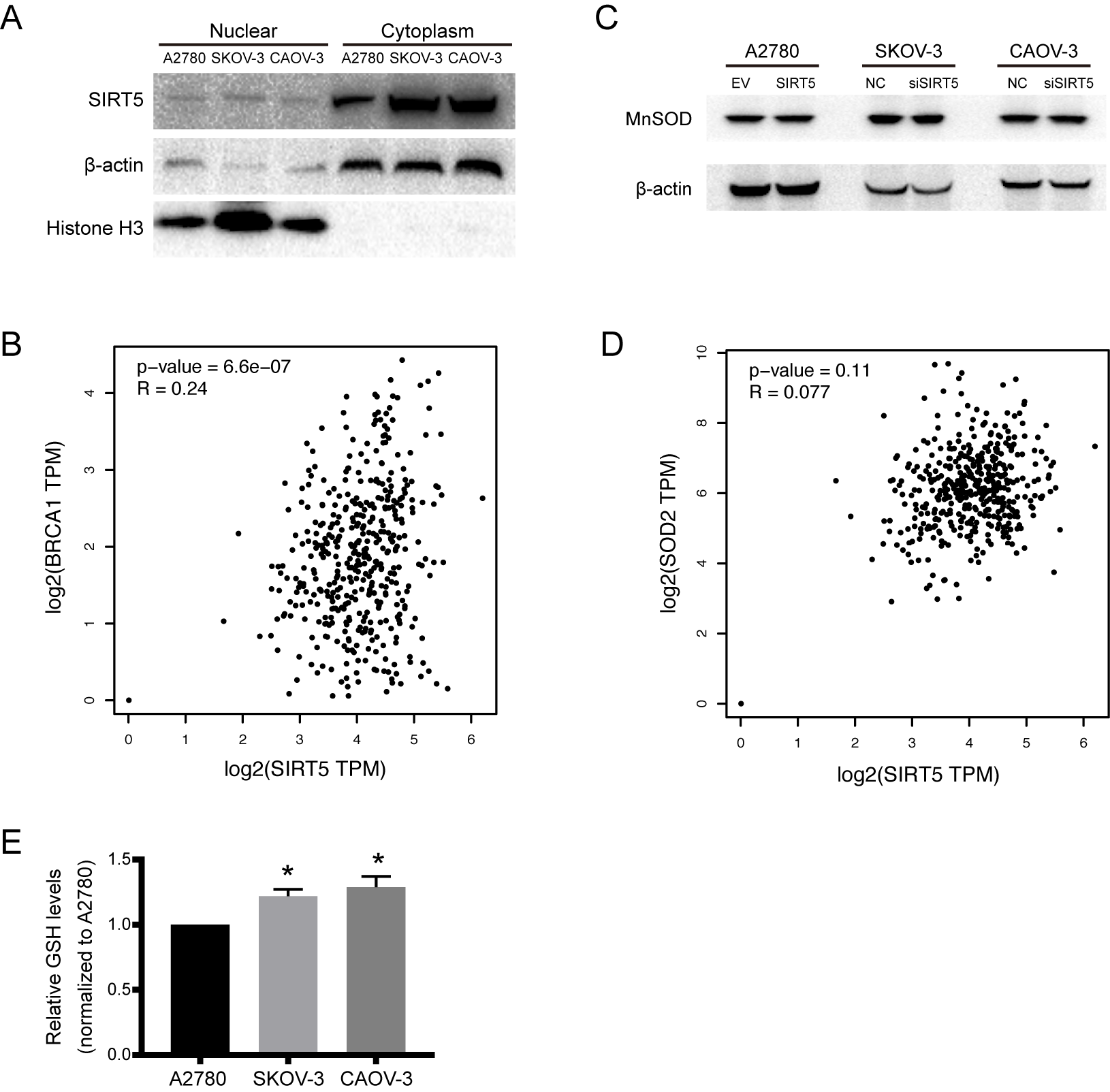

Supplement: Figure S1 — (A) The localization of SIRT5 in three ovarian cancer cell lines was validated by western blot after nuclear and cytoplasm isolation. (B) SIRT5 had a positive relationship with BRCA1 based on GEPIA database. (C) MnSOD/SOD2 protein levels were not significantly changed after upregulation or downregulation of SIRT5 levels. (D) SIRT5 had no significant relationship with MnSOD/SOD2 based on GEPIA database. (E) The relative levels of glutathione (GSH) in three ovarian cancer cells. TPM, transcripts per million. *P < 0.05. [file Image_1.TIF]
